# Evaluation of Dietary Assessment Tools Used in Bariatric Population

**DOI:** 10.3390/nu13072250

**Published:** 2021-06-29

**Authors:** Marianne Legault, Vicky Leblanc, Geneviève B. Marchand, Sylvain Iceta, Virginie Drolet-Labelle, Simone Lemieux, Benoît Lamarche, Andréanne Michaud

**Affiliations:** 1 Quebec Heart and Lung Institute Research Centre, Université Laval, Québec City, QC G1V 4G5, Canada; marianne.legault.2@ulaval.ca (M.L.); sylvain.iceta.1@ulaval.ca (S.I.); virginie.drolet-labelle.1@ulaval.ca (V.D.-L.); 2 Centre Nutrition, Santé et Société (NUTRISS), Institut sur la Nutrition et les Aliments Fonctionnels (INAF), Université Laval, Québec City, QC G1V 0A6, Canada; vicky.leblanc@fsaa.ulaval.ca (V.L.); genevieve-b.marchand.1@ulaval.ca (G.B.M.); simone.lemieux@fsaa.ulaval.ca (S.L.); benoit.lamarche@fsaa.ulaval.ca (B.L.)

**Keywords:** obesity, dietary assessment tool, 24 h dietary recall, food frequency questionnaire, food record, bariatric surgery

## Abstract

Severe obesity is associated with major health issues and bariatric surgery is still the only treatment to offer significant and durable weight loss. Assessment of dietary intakes is an important component of the bariatric surgery process. Objective: To document the dietary assessment tools that have been used with patients targeted for bariatric surgery and patients who had bariatric surgery and explore the extent to which these tools have been validated. Methods: A literature search was conducted to identify studies that used a dietary assessment tool with patients targeted for bariatric surgery or who had bariatric surgery. Results: 108 studies were included. Among all studies included, 27 used a dietary assessment tool that had been validated either as part of the study per se (*n* = 11) or in a previous study (*n* = 16). Every tool validated per se in the cited studies was validated among a bariatric population, while none of the tools validated in previous studies were validated in this population. Conclusion: Few studies in bariatric populations used a dietary assessment tool that had been validated in this population. Additional studies are needed to develop valid and robust dietary assessment tools to improve the quality of nutritional studies among bariatric patients.

## 1. Introduction

Obesity is a common, complex chronic disease and its prevalence has increased over the past several years, making it a major public health concern [1]. More importantly, the prevalence of severe obesity (BMI ≥ 35 kg/m^2^) has increased dramatically in Canada [2]. Severe obesity is associated with major health issues such as an increased risk of hypertension, type 2 diabetes, sleep apnea and cancer [2]. Bariatric surgery is the only treatment for severe obesity to offer significant and durable weight loss as well as improvement of metabolic diseases [3]. Multiple types of surgery exist and are usually classified as restrictive, malabsorptive or mixed-procedures. Restrictive surgery limits the amount of food consumed by reducing stomach size, while malabsorptive surgery limits nutrient absorption by bypassing or reorganizing parts of the small intestine. Mixed-procedures, the most common surgeries, combine both gastric restriction and intestinal malabsorption [4,5]. Assessment of dietary intakes and eating behaviors are important components of the bariatric surgery process especially after surgery, since diet quality of bariatric patients is most likely to impact their risk of developing nutritional deficiencies [6] and their food preferences and choices could impact the success of their weight loss [7]. 

Many dietary assessment tools are used in nutritional research, especially self-report tools because they are often easier to use and less expensive as opposed to using controlled feeding environments, direct observation or measurements of biomarkers. The most common self-reported tools are food records (FR), 24-h dietary recalls (24HR) and food frequency questionnaires (FFQ). Over the last years, these tools have been adapted for a web-based use, such as self-administered web-based 24HR [8,9] or a web-based FFQ [10,11], to increase cost-efficiency and therefore the applicability to large cohort studies. The FR is a dietary assessment tool where respondents have to report all the foods and beverages consumed during the current day with as many details as possible (portion size, brand, method of cooking, time of the day, location of the eating occasion, etc.), for a variable number of days (often between 3 and 7) [12]. The 24HR consists of listing detailed information about everything the respondent ate and drank from midnight to midnight the previous day, or over the past 24-h period [12]. Finally, FFQ is a fixed-sequence questionnaire based on a predetermined series of foods and beverages consumed over a given period of time, which can be the previous week, month or year. The number and size of portions are often asked subsequently [12]. Of all these dietary assessment tools, the 24HR has been hypothesized as the least biased dietary assessment tool, since FR is more associated to reactivity biases such as a tendency to modify the usual diet for a more socially desirable manner or to simplify the recording task, and FFQ is known to encompass more important systematic biases than 24HR (does not capture the entire diet due to difficulty of the recall task) [12]. 

One reason explaining the difficulty to select the most appropriate dietary assessment tool with patients targeted for bariatric surgery and patients who had bariatric surgery is the relative lack of validation of these tools within those specific populations. Validity of an instrument is the degree to which an instrument measures what it is supposed to measure [12]. To determine the validity of an instrument, it is often compared with another instrument measuring the same concept and known to be accurate or considered as a gold standard [12]. Validation of dietary assessment is conducted to determine how accurately self-report instruments measure true dietary intakes [13]. It is crucial to develop and use tools that provide an accurate and precise measure of dietary intakes to optimize treatment and the nutritional care provided to patients targeted for or who had bariatric surgery [6,14]. Moreover, as patients who have undergone bariatric surgery have a higher risk of developing nutritional deficiencies [6], it is also essential to select dietary assessment tools validated for global intakes, particularly protein intakes since it is the major macronutrient deficiency after bariatric surgery [14,15]. 

The aim of this review was to document the dietary assessment tools that have been used in research involving patients targeted for bariatric surgery and patients who had bariatric surgery, and to explore the extent to which these tools have been validated.

## 2. Methods

### 2.1. Search Strategy

A literature search was conducted for all articles published on Pubmed up to January 2021 to identify studies that used dietary assessment tool with patients targeted for bariatric surgery or who had bariatric surgery. The search strategy was done using this keywords combination: “food intake”[All Fields] OR “food intake evaluation”[All Fields] OR “dietary intake”[All Fields] OR “dietary intake evaluation”[All Fields] OR “dietary assessment”[All Fields] OR “dietary assessment evaluation” [All Fields] OR “food assessment” [All Fields] OR “food assessment evaluation” [All Fields] AND bariatric [All Fields]. 

### 2.2. Selection of Studies

The literature search was performed independently by three authors (G.B.M., M.L., V.L.) and included all studies published on Pubmed up to 2021. Studies were found and retained in three stages: (i) the first stage was a screening done directly on Pubmed according to the title and abstract, (ii) the second one was the complete reading of the articles, and (iii) the third stage was a screening of the references of the retained articles. Inclusion and exclusion criteria that were used are presented in Table 1. Only original studies were included in this review, based on the inclusion and exclusion criteria. 

### 2.3. Data Extraction

The following data were extracted by three authors (M.L., G.B.M., V.D.-L.) for each study: (a) bibliographical data (author, publication year, country); (b) sample characteristics (sample size, type of surgery, mean and standard deviation (SD) for age, sex and body mass index); (c) study design features (objective, study design and dietary assessment tool); (d) outcomes (self-reported energy and nutrient intakes, information on the validity of the dietary assessment tool, if available) (Table 2). Information regarding the validity of the dietary assessment tool was also extracted, such as the reference method used for validation, the population in which the validation has been performed, and information about the validation process (Table 3).

## 3. Results

### 3.1. General Overview

**Figure 1:**
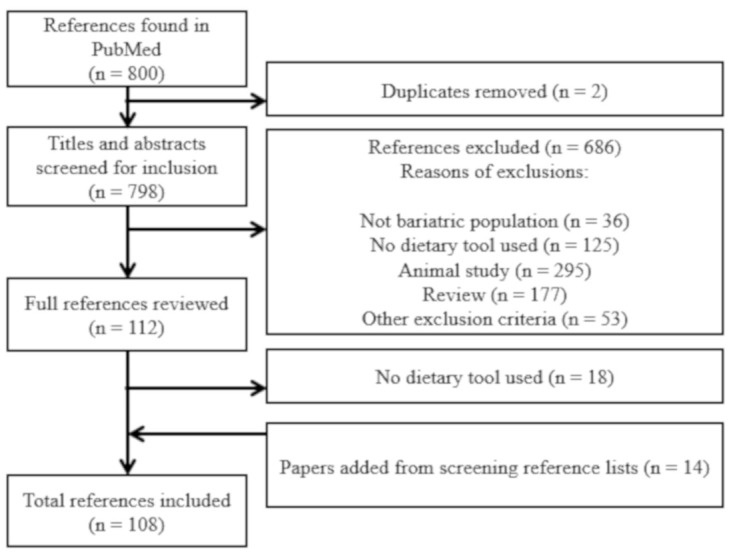
Flowchart for selection of included references.

As shown in Figure 1, a total of 800 references were generated by the search strategy in Pubmed, and 108 original studies were included in this review by fulfilling our inclusion and exclusion criteria. Table 2 shows an exhaustive description of the 108 studies included. Studies were published between 1989 and 2021 and were conducted in many countries. These studies represented a total of 10 046 participants (74% females). Twenty five studies (23%) included more than 100 participants, including one with 1695 participants. The mean BMI was 46.0 kg/m^2^ (between 29.2 and 55 kg/m^2^) with a mean age of 44 years (between 33 and 65 years old). Among studies, 75 (69%) included Roux-en-Y gastric bypass (RYGB), 26 (24%) sleeve gastrectomy (SG), 19 (17%) gastric banding (GB) and 4 (4%) biliopancreatic diversion (BPD). Thirty-three (31%) studies included more than one type of surgery, and 8 (7%) studies did not specify the type of surgery performed. Almost all studies were classified as prospective (*n* = 32), cross-sectional (*n* = 26), retrospective (*n* = 22) or longitudinal (*n* = 18).

**Figure 2:**
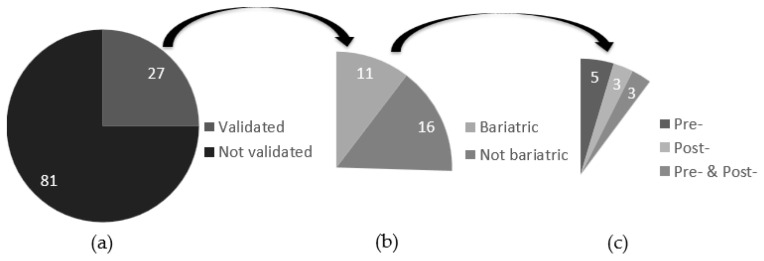
(**a**) Number of tools validated and not validated; (**b**) Number of tools validated within a bariatric population; (**c**) Number of tools validated pre- and post- bariatric surgery.

To assess dietary intakes, 38 studies used FR [16,19,20,21,24,27,31,36,39,41,42,43,46,51,52,53,57,58,59,61,62,63,68,70,72,74,80,81,91,94,99,100,105,107,111,112,118,121], 32 used 24HR [23,25,32,35,37,44,45,49,60,64,67,73,77,78,79,85,86,87,88,89,90,92,95,96,97,98,106,110,115,119,122,123], 16 used FFQ [18,26,40,48,50,65,76,84,101,102,103,104,108,109,114,117], 8 used questionnaires (6 were inspired by FFQ [29,66,69,71,93,120] and 2 did not provide details [22,55]), 2 used other dietary assessment methods (photo-assisted capture method and food and symptom diary) [17,75], and 12 studies used combined tools [28,30,33,34,38,47,54,56,82,83,113,116] (Table 2). Among all studies included, 27 used a dietary assessment tool that had been validated either as part of the study per se (*n* = 11) or in a previous study (*n* = 16) (Figure 2). Table 3 presents the 27 studies included in this review that used a validated dietary assessment tool. Every tool validated per se in the cited studies was validated among a bariatric population, while none of the tools validated in previous studies were validated in this population. Among the 11 studies, 3 validated their tool pre- and post-surgery, 5 validated it only pre-surgery and 3 post-surgery only (Figure 2).

### 3.2. Validation of Dietary Assessment Tools in Bariatric Population

#### 3.2.1. Food Records (FR)

Of the three studies having tested the validity of the FR per se in their bariatric population, two studies [27,63] used indirect calorimetry as a reference and one study [121] used plasma concentrations biomarkers (vitamin A, D, E and C) as reference (Table 3). Regarding the validity of the tools, Bobbioni-Harsh et al. [27] found that the mean self-reported energy intake from their 3-day FR was 17.2% lower than energy requirement evaluated with indirect calorimetry pre-surgery. Golzarand et al. [63] found that protein and carbohydrate oxidation were significantly decreased post-surgery. Wolf et al. [121] found no correlation between self-reported dietary intakes obtained from a 3-day FR pre-surgery and corresponding serum concentrations biomarkers of intake (25-hydroxycholecalciferol, retinol, ascorbic acid, tocopherol/cholesterol ratio, β-carotene, calcium, magnesium, phosphate).

#### 3.2.2. 24-h Dietary Recall (24HR)

Four studies tested the validity of the 24HR *per se* in their bariatric population [67,92,97,115] (Table 3). In two of those studies, indirect calorimetry (resting metabolic rate, energy requirement) pre-surgery [97] and pre- and post-surgery [115] was used as a reference. Total daily energy intake assessed by 24HR was below measured resting metabolic rate pre-surgery by 8% in Verger et al.’ study [115], while Quesada et al. [97] found that 55 to 97% of their participants underreported their intake compared to resting metabolic rate. Another study [67] tested the validity of their 24HR using 24-h urine recovery biomarker data as a reference for protein intake pre-surgery, and another one [92] used FR post-surgery as a reference (energy, macro and micronutrient intakes). Kops et al. [67] concluded that approximately 37% of bariatric patients underreported protein intakes pre-surgery assessed with 24HR compared to 24-h urinary recovery biomarker data, while 25% overreported it. Novais et al. [92] validated their 24HR by comparing it with a 3-day FR and found a high level of agreement between both tools for energy and nutrient intakes.

#### 3.2.3. FFQ

One study [34] directly tested FFQ validity using a 24HR as a reference in post-surgery patients and found a difference of 150 kcal between the two methods (1230 kcal with the FFQ vs. 1083 kcal with the 24HR) (Table 3). 

#### 3.2.4. Questionnaires 

None of the studies that used a questionnaire to assess mean daily energy intake used a questionnaire validated in bariatric population. It is important to mention that little information was available about the form of questionnaires used. Five studies [66,69,71,93,120] used the Swedish Obese Subjects (SOS) study questionnaire [132] (Table 3), which was adapted from a simplified dietary history interview and was previously validated using a 4-day FR, nitrogen urinary excretion and 24 h energy expenditure measured by indirect calorimetry in obese and non-obese population, but not in bariatric population. 

#### 3.2.5. Other Dietary Assessment Methods

Al-Ozairi et al. [17] used a photo-assisted diet capture method to assess energy intake in post-surgery (Table 3). They found that after SG, patients reported a higher energy intake with the 24HR compared to estimations obtained using photographs, but they suggested that digital photography was more reliable and accurate for measuring energy intake in this specific population than 24HR [17]. 

#### 3.2.6. Mixed Methods

Two studies validated the use of mixed methods to assess dietary intakes among bariatric population [28,33] (Table 3). Casagrande et al. [33] used both FFQ and 24HR to assess dietary intakes pre-surgery. Protein, cholesterol and sodium intakes were lower with the FFQ than with the 24HR, while calcium intake was higher [33]. To assess the accuracy of the estimated mean dietary intake found with the 24HR, Brolin et al. [28] used a 1-week FFQ to compare both dietary intakes pre-surgery. They found statistically significant correlations between the tools for total energy intake and intake of milk and ice cream products, sweet/soda and nonliquid sweets [28]. 

## 4. Discussion

The objective of this review was to document the dietary assessment tools used among patients targeted for bariatric surgery and those who have undergone bariatric surgery. A total of 108 studies were included in this review; only 27 (25%) validated their dietary assessment tool or used a tool that had been previously validated, and only 11 (10%) were validated in bariatric population. Of these 11 studies, only 3 of them validated the dietary assessment tool before and after surgery, 5 validated it only before surgery, and 3 only after surgery.

The validation process of dietary assessment tools is complex but is imperative in order to evaluate usual dietary intakes and also provide an adequate estimation of nutrient intakes and potential deficiencies following bariatric surgery [6]. As previously mentioned, the dietary assessment tool of interest is often compared with another tool measuring the same concept and known to be accurate or considered as a gold standard to determine the validity [3,4]. Direct observation, which refers to objective assessment of foods and beverages consumed, is also frequently used in a clinical setting [5]. This method remains the best option to exclude risk of estimation bias, which could be present with another dietary assessment tool [2], but it is not representative of usual intakes and can cause other biases such as response bias since participants are being observed. No study using direct observation were found for this review. Most of validation studies included in this review used the comparison with another dietary assessment tool (*n* = 4; 1 FR, 2 24HR, 1 FFQ) or used indirect calorimetry (*n* = 4) to assess energy expenditure and macronutrient’s oxidation. Indirect calorimetry is less biased than self-report dietary assessment tools [12], however the later are more commonly selected as they are more accessible [12]. In the general population, FR are the most commonly used self-report tools to validate dietary intakes [8]. In order to improve quality of the validation process, the dietary assessment tool needs to be tested and compared, by direct observation or with a reference method, within the same population [6,7]. In the current review, we found that only 10% of the validated tools were validated in a bariatric population, showing a clear lack of studies that used a tool validated in that specific population. Moreover, conclusions about validity of the tools varied considerably among studies (as seen in Table 3). However, in general, FR were found as acceptable as a dietary assessment tool [27,124]. Authors found underreporting of dietary intake while validating their 24HR [97,115], but it was still deemed appropriate [67,92], particularly when used within epidemiological studies [125]. Studies examining the validation of FFQs found almost the same conclusion, namely more accurate with groups than individuals [127,129] and with a reasonable validity [128,130,131]. The only validated questionnaire had the tendency to report higher dietary intake than FR or nitrogen excretion [132]. Finally, digital photography seems to be a reliable and accurate tool for dietary intakes assessment [17], but more studies are needed to confirm these results. 

Factors characterizing the bariatric population such as bias and stigmatization, dietary requirements pre- versus post-surgery and type of surgery might influence the choice of the dietary assessment tool and need to be considered in the validation process. Inclusion of patients who will have bariatric surgery and patients who have undergone bariatric surgery in the same study can be questioned as characteristics of patients and susceptible biases in reporting dietary intakes can broadly differ. For instance, social desirability biases and stigmatization can be stronger prior to than after bariatric surgery [133] since patients want to be eligible for the surgery and do not want to be excluded based on some inadequate eating habits. In addition, because several types of bariatric surgeries exist and have different impact on energy restriction and nutrient’s absorption, the need to categorize individuals according to the type of surgery, more specifically post-surgery, should also be considered in the validation process. Some studies included in this review evaluated a cohort longitudinally and assessed dietary intakes pre- and post-surgery using the same dietary assessment tool, but none of them differentiated the validity of the tool to measure dietary intakes prior to and after surgery.

This review has strength and limitations. It showed an important lack of studies that used a tool validated in bariatric population and the need to conduct research to address this concern. Indeed, a considerable number of studies used a dietary assessment tool that had been previously validated in a non-bariatric population, such as the Swedish Obese Subjects study questionnaire. Furthermore, only a few studies included in this review specifically aimed to validate the dietary assessment tool used to assess dietary intakes in bariatric population, another indicator of the lack of literature. The interpretation of the results remained difficult considering the limited availability of information regarding the validation process and conclusions about the validity in most studies, and the high level of methodological differences between studies. 

Identification of the most relevant dietary assessment tools validated prior to and after bariatric surgery would allow to characterize dietary intakes more accurately while improving nutritional interventions among these patients. Validity of dietary assessment tools should be tested for total daily energy intake and in terms of diet quality. Indeed, quality of dietary intakes of patients targeted for bariatric surgery can impact their risk of developing nutritional deficiencies after the surgery [6] and the success of their weight loss [93]. Moreover, web-based and technology-assisted assessment methods have opened the way to a new wave of self-administered automatic tools [8,9]. Considering that the web-based 24HR has been associated with reduced desirability bias compared to standard administrated questionnaires at least in the general population [8], such tools could be an interesting approach to assess dietary intake in bariatric population. The potential benefits and risks associated with these web-based tools need to be evaluated in bariatric population. More studies about the validation of dietary assessment tools in bariatric population are needed, taking into account potential biases in this population.

## 5. Conclusions

In conclusion, few studies included in the review validated their dietary assessment tool. Additional studies are needed in order to develop valid and robust dietary assessment tools among bariatric population. These tools are essential in evaluating efficacy of nutritional interventions conducted in this population.

## Figures and Tables

**Table 1:** Inclusion and exclusion criteria.

Inclusion Criteria	Exclusion Criteria
(i) Population: *n* ≥10 (ii) Adult population (iii) Bariatric population (pre and post surgery) (iv) Use of a dietary assessment tool (v) Original research in English or French	(i) Animal studies (ii) Studies evaluating disorders only (iii) Case report (iv) Review (v) Studies analyzing a cohort already included in the present review (vi) Studies related to pregnancy

**Table 2:** Exhaustive description of included articles.

Study	Year	Country	PMID	Population				Dietary Assessment		Surgery Type	Objective	Study Design
				N	Age ± SD	Sex	BMI ± SD	Tool	Validation			
Al Assal et al. [16]	2020	Brazil	31973130	25	45.8 ± 7.9	100% women	46.4 ± 5.5	Food record (7 days)	None	RYGB	Assess the gut microbiota profile before and after RYGB and the correlation with food intake and postoperative type 2 diabetes remission.	Prospective
Al-Ozairi et al. [17]	2019	Kuwait	30756296	50	38.8 ± 9.1	84% women	29.2 ± 6.2	Photo-assisted diet capture method	Yes	SG	Evaluate the use of digital food photography in comparison to conventional methods among patients after sleeve gastrectomy.	Cross-sectional
Amundsen et al. [18]	2017	Norway	27914028	49	46	82% women	44.1	FFQ	Yes	GB	Compare GB patients experiencing suboptimal weight loss or significant weigh regain with successful controls, regarding postoperative food intake, eating behavior, physical activity, and psychometrics.	Case-control
Andersen and Larsen [19]	1989	Denmark	2556911	18	35	89% women	N/D	Food record (7 days)	None	Gastroplasty	Evaluate diet compliance and nutritional safety.	Longitudinal
Anderson et al. [20]	2007	United-states	17557983	84	AA: 41 ± 10; white: 43 ± 10	76% women	AA: 55 ± 10; white: 53 ± 11	Food record (N/D)	None	RYGB	Compare weight loss between AA and white severely obese patients after RYGB and examined differences in dietary intake and cardiovascular risk factors before and after weight loss.	Retrospective
Andreu et al. [21]	2010	Spain	20820937	101	43.2	75% women	47.7	Food record (3 days)	None	RYGB (66%) or SG (34%)	Examine the accomplishment of the recommended protein intake, and the influence of protein intake on free fat mass and protein status following bariatric surgery.	Longitudinal
Anthone et al. [22]	2003	United States	14530733	701	42.3 ± 10.4	78% women	52.3 ± 9.6	Questionnaire	None	DS	Determine the safety and efficacy of the duodenal switch procedure as surgical treatment of morbid obesity.	Prospective
Aron-Wisnewsky et al. [23]	2016	France	26891123	22	GBP: 40.5; AGB 40.5	100% women	GBP 46.3; AGB 42.8	3 × 24-h dietary recall	Yes	GBP or AGB	Analyze the effect of food restriction on nutritional parameters in the short-term (≤3 months) period after bariatric surgery in morbid obesity.	Prospective
Barnadas et al. [24]	2021	Spain	33435751	41	49.7 ± 10	75% women	44.3 ± 6.2	Food record (7 days)	None	SG	Study the alterations of the circadian rhythmicity due to morbid obesity and the recovery of the circadian pattern after weight loss in a cohort of patients who underwent sleeve gastrectomy.	Prospective
Bavaresco et al. [25]	2010	Brazil	18931884	48	41.9	85% women	51.9	24-h dietary recall	None	RYGB	Assess the metabolic and nutritional profile of grade III obese patients for a period of 12 months after bariatric surgery.	Longitudinal
Benaiges et al. [26]	2019	Spain	31288988	60	43.1 ± 7.9	71.7% women	44.1 ± 4.9	FFQ	Yes	RYGB (43%)	Evaluate dietary modifications during the preoperative and postoperative periods of bariatric surgery.	Observational, prospective
Bobbioni-Harsch et al. [27]	2002	Switzerland	12032656	50	38.4	100% women	45.2	Food record (3 days)	Yes	RYGB	Evaluate whether or not the individual differences in the substrates content of the diet had any impact on body weight loss and, consequently, could contribute to its variability.	Longitudinal
Brolin et al. [28]	1994	United-states	7986146	138	VBG: 39 ± 9; RYGB: 38 ± 10	85% women	VBG: 42 ± 4; RYGB: 43 ± 4	Dietary history + 24-h dietary recall	Yes	VBG (30) or RYGB (108)	Determine whether assessment of preoperative eating patterns and food preferences can be used to predict weight loss outcome after surgery.	Prospective longitudinal
Buzga et al. [29]	2014	Czech Republic	25561993	37	43.5	78% women	43	Questionnaire	None	SG	Assess changes in dietary habits in obese patients 6 and 12 months after SG, compare changes in hormonal levels and dietary habits after this procedure.	Longitudinal
Carrasco et al. [30]	2007	Chile	17658019	38	36.3	89% women	44	FFQ + Food record (3 days)	None	RYGB	Detect metabolic or behavioral parameters that could predict the reduction in weight, the loss in body fat and the improvement in cardiovascular risk factors.	Longitudinal
Carrasco et al. [31]	2012	Chile	22305536	50	37.6 ± 10.2	100% women	43.8 ± 4.8	Food record (3 days)	None	GBP	Evaluate the relation between weight loss and food intake and between weight loss and changes in serum ghrelin concentrations 1 y after GBP with resection of the bypassed stomach and without resection.	Prospective
Carvalho et al. [32]	2020	Brazil	32728839	122	33	77% women	N/D	2 × 24-h dietary recall	Yes	RYGB or SG	Evaluate the association between social jet lag, a measure of circadian misalignment, and anthropometric, metabolic and food intake outcomes 6 months after bariatric surgery.	Longitudinal, observational
Casagrande et al. [33]	2010	Brazil	20411350	33	35.9	100% women	43.2	FFQ + 24-h dietary recall	Yes	RYGB	Evaluate bone metabolism/mineral density and nutritional profile in morbidly obese women before surgery.	Prospective longitudinal
Chou et al. [34]	2017	Taiwan	28589529	40	33.5 ± 9.7	75% women	37.9 ± 6.6	FFQ + 24-h dietary recall	Yes	SG	Investigate long-term dietary intake and weight status after SG.	Retrospective
Coluzzi et al. [35]	2016	Italy	26744284	30	35	73% women	43.9	24-h dietary recall	None	SG	Evaluate the quantitative reduction and qualitative changes in food intake post surgery and analyzed the association between weight loss and changes in eating behavior.	Prospective longitudinal
Cooper et al. [36]	1999	Australia	10340816	26	23–59	96% women	31.6–52.7	Food record (4 days)	None	MLVG	Perform detailed dietary analyses together with anthropometric, haematological and food intolerance assessment of a group of subjects undergoing MLVG, who received preoperative dietary education and regular postoperative followup, with some dietary advice over the subsequent year.	Longitudinal
Correia Horvath et al. [37]	2014	Brazil	24528344	77	44.5	65% women	48.8	24-h dietary recall	None	N/D	Assess food consumption by severely obese patients and describe their main nutritional deficiencies on the basis of dietary reference intake.	Cross-sectional
Coupaye et al. [38]	2014	France	24122661	86	SG: 45 ± 11; RYGB: 44 ± 9	72% women	SG = 48.5 ± 9.6; RYGB = 48.6 ± 7.8	Food record (4 days) + interview	None	RYGB or SG	Compare nutritional status after SG and RYGB in subjects matched for postoperative weight	Prospective
Custodio et al. [39]	2012	Brazil	23165553	22	37.9 ± 9.1	100% women	44.3 ± 5.4	Food record (3 days)	None	RYGB	Evaluate the influence of changes in food intake on body composition and some hematologic and biochemical variables in the period of eight weeks after RYGB.	Prospective
Dagan et al. [40]	2016	Israel	26797718	100	41.9	60% women	42.3	FFQ	None	SG	Evaluate and compare between genders dietary intake and micronutrient deficiencies among 100 candidates for surgery.	Cross-sectional
Dagan et al. [41]	2017	Israel	28303504	77	43.1	57% women	42.1	Food record (3 days)	None	SG	Evaluate adherence to dietary and lifestyle recommendations and its relation to weight post surgery.	Prospective
Davies et al. [42]	2020	New Zealand	32447634	44	RYGB: 48.5 ± 5.5 SG: 47.7 ± 6.9	52% women	RYGB: 38.2 ± 5.7; SG: 40.0 ± 5.9	Food record (5 days)	None	RYGB or SG	Identify whether there were surgery-specific changes in gut microbiota among obese people with Type 2 diabetes randomised to either SG or RYGB and whether there were common taxa and gut microbiota functional capacity changes among those who achieved T2D remission, irrespective of surgery type.	Prospective
da Silva et al. [43]	2014	Brazil	25518027	10	46.5 ± 6.6	100% women	45.7 ± 4.1	Food record (7 days)	N/D	RYGB	Compare the Virtual Nutri Plus^®®^ and Dietpro 5i^®®^ software systems in assessing nutrient intake in obese patients with type 2 diabetes mellitus who underwent a RYGB.	Prospective
da Silva et al. [44]	2016	Brazil	27544005	80	46	88.8% women	49.8 ± 9.3	2 × 24-h dietary recall	None	RYGB	Investigate factors associated with weight regain long after RYGB.	Retrospective
Dias et al. [45]	2006	Brazil	16680324	40	42.5 ± 10.8	100% women	51.9 ± 11.8	24-h dietary recall	None	RYGB	Systematically document nutrient intake at 3-month intervals, during the first 12 months after uncomplicated RYGB.	Prospective
Duffey et al. [46]	2007	United States	18289566	45	47.0 ± 10.5	71% women	49.5 ± 9.1	Food record (1 day)	None	N/D	Evaluate baseline risk factors for stone formation in a group of morbidly obese patients presenting for gastric bypass surgery and the changes that may occur after bariatric surgery.	Cross-sectional
El Labban et al. [47]	2015	Lebanon	25982803	60	RYGB: 39.6 ± 11.3; SG: 33.0 ± 12.3	60% women	RYGB: 42.7 ± 5.2; SG: 41.2 ± 4.1	FFQ + 3 × 24-h dietary recall	Yes	RYGB or SG	Compare dietary intake, food preferences, and gastro-intestinal symptoms in subjects with extreme obesity after RYGB and SG.	Cross-sectional
Ernst et al. [48]	2009	Germany	19034589	121	RYGB: 40.2 ± 1.5; GB: 44.0 ± 1.2	79% women	RYGB: 46.5 ± 0.7; GB: 44.6 ± 0.5	FFQ	None	GBP (48) or GB (73) + 45 obese controls	Assesse dietary habits in patients who have underwent a bariatric surgery and compare their data with those of an obese as well as a nonobese control group.	Cross-sectional
Faria et al. [49]	2014	Brazil	25409965	60	N/D	87% women	N/D	3 × 24-h dietary recall	None	RYGB	Compare weight loss, consumption of macronutrients and the frequency of vomiting among patients who underwent RYGB with and without the placement of a constriction ring around the pouch.	Retrospective
Farias et al. [50]	2020	Brazil	32200267	32	40.1 ± 10.1	94% women	43.9 ± 1.1	FFQ	Yes	RYGB	Analyze the contribution of unprocessed, processed, and ultra-processed foods 2 years after RYGB.	Prospective
Federico et al. [51]	2016	Italy	27107092	28	26–63	71% women	Women: 48.6 ± 8.1; Men: 54.3 ± 18.5	Food record (7 days)	None	BI	Evaluate the dietary intake, the nutritional status, as well as plasma levels of a number of gastrointestinal peptides that regulate food intake and fecal microbiota in severely obese patients and healthy non-obese control subjects and evaluate whether bariatric surgery affected gastrointestinal peptides plasma levels and fecal microbiota.	Prospective longitudinal
Forbes et al. [52]	2016	United-states	26328533	18	36.6 ± 2.3	100% women	44.0 ± 1.0	Food record (3 days)	None	RYGB (13) or AGB (5)	Describe compositional changes in plasma phospholipids during 6 months following bariatric surgery procedures.	Longitudinal
Freeth et al. [53]	2012	United-states	22714824	15	18–80 (min-max)	N/D	N/D	Food record (3 days)	None	RYGB (6) or GB (9)	Comprehensively analyze selenium intake before and after bariatric surgery while simultaneous looking at the serum selenium level and functional measurement of selenium.	Prospective longitudinal
Freeman et al. [54]	2013	Australia	24743015	130	Control: 47; AGB 46; RYGB 58; SG 50	68% women	Control 43.2; AGB 45.5; RYGB 42.4; SG 43.2	Questionnaire + 24-h dietary recall	None	AGB, SG or RYGB	Assess food tolerance and diet quality in AGB, SG and RYGBP patients 2–4 years post-surgery, comparing findings with an obese control group.	Prospective, cross-sectional
Furet et al. [55]	2010	France	20876719	30	nDb: 42 ± 2; Db: 49 ± 5	90% women	nDb: 48.3 ± 1.6; Db: 45.4 ± 3.5	Questionnaire	None	RYGB	Examine the association between gut microbiota changes and a range of body composition, metabolic, and inflammatory markers.	Prospective longitudinal
Furtado et al. [56]	2018	Brazil	30307293	105	Succes group 43.3 ± 11.4; Failure group 43.4 ± 10.7	84% women	SG 48.8 ± 8.4; Failure group 49.9 ± 6.6	24-h dietary recall + food record (3 days) + FFQ	None	RYGB	Analyse wheter feeding behavior, evaluated by caloric intake, dietary preferences and tolerance, can be considered as a determinant factor for weight loss in obese patients submitted to RYGB.	Cross-sectional
Gesquiere et al. [57]	2017	Belgium	27591033	54	48	61% women	40.4	Food record (2 days)	None	RYGB	Study dietary and supplement intake of micronutrients before and after RYGB and examine the association between the total micronutrient intakes and status markers.	Prospective longitudinal
Gimenes et al. [58]	2017	Brazil	28102495	25	35.7	100% women	50.1 ± 6.5	Food record (1 day)	None	RYGB	Evaluate nutritional and biochemical indicators of women who became pregnant after RYGB.	Retrospective
Giusti et al. [59]	2015	Switzerland	26675775	16	39.4 ± 2.4	100% women	44.1 ± 1.6	Food record (7 days)	None	RYGB	Evaluate energy and macronutrient intakes, body composition, and the basal metabolic rate in obese female patients during the initial 3 y after an RYGB.	Observational
Gobato et al. [60]	2014	Brazil	25264334	36	37.7	75% women	44.2	24-h dietary recall	None	RYGB	Evaluate the nutritional status of minerals and vitamins and the food consumption in patients before and after RYGB. Evaluate the overall effect of RYGB by monitoring additional risk factors of related chronic diseases.	Prospective longitudinal
Gobato et al. [61]	2018	Brazil	30306500	75	38 ± 10	89% women	43.94 ± 5.89	Food record (3 days)	None	RYGB	Evaluate the food intolerance after banded RYGB, correlating the data of food ingestion.	Observational, prospective
Golpaie et al. [62]	2011	Iran	22266100	30	32.5	70% women	44.1 ± 4.9	Food record (3 days)	None	AGB (15) or (16) TGVP	Investigate the possible short-term effects of surgery on vaspin and other metabolic variables relevant to insulin sensitivity and evaluate the possible relationship between dietary intake and serum vaspin.	Longitudinal
Golzarand et al. [63]	2018	Iran	30251098	43	N/D	N/D	RYGB: 45.9 ± 4.6 SG: 39.5 ± 4.2	Food record (3 days)	Yes	RYGB or SG	Compare the changes in body composition, dietary intake, and substrate oxidation 6 months post-sugery in obese patients who underwent RYGB and SG.	Prospective
Jastrzębska-Mierzyńska et al. [64]	2012	Poland	23256020	27	Women: 40.4 ± 13.9; Men: 39.6 ±12.7	68% women	W: 45.9 ± 6.8; M: 48.1 ± 7.7	24-h dietary recall	None	N/A	Assess dietary habits, nutritional status and biochemical parameters of blood in patients being prepared for different bariatric procedures.	Cross-sectional
Johnson et al. [65]	2013	Norway	23110916	72	42.6 ± 11	69% women	46.2 ± 5.9	FFQ	Yes	RYGB	Compare changes in the dietary patterns of morbidly obese patients undergoing either laparoscopic gastric bypass surgery or a comprehensive lifestyle intervention program.	Interventional (clinical trial)
Kanerva et al. [66]	2017	Sweden	28756049	1695	47.3 ± 5.9	69.8% women	42.5 ± 4.5	Questionnaire	Yes	LAGB OR VBG OR RYGB	Explore whether pre-surgical sociodemographic and lifestyle characteristics, together with the type of surgery, could predict 10-year changes in dietary intake following bariatric surgery.	Prospective, matched, non-randomized, surgical intervention trial
Kops et al. [67]	2017	Brazil	28760427	120	N/D	Adherent: 74.4% women; Non adherent 81.8% women	Adherent: 45.8 ± 6.8 Non adherents: 49.1 ± 8.1	24-h dietary recall (3×)	Yes	N/D	Evaluate the possible association between the Thr54 allele and anthropometric and lipid profile of severely obese indivieuals, taking into account the dietary intake of these participants.	Cross-sectional
Kruseman et al. [68]	2010	Switzerland	20338278	141	40	93% women	46	Food record (4 days)	None	GBP	Document weight and body composition changes among patients after bariatric surgery and to assess whether dietary, behavior, or psychological factors were associated with long-term weight outcome.	Retrospective longitudinal
Laurenius et al. [69]	2013	Sweden	23299713	43	43 ± 10	72% women	44.3 ± 4.9	Questionnaire	Yes	RYGB	Test the hypothesis that dietary energy density decreases after RYGB.	Longitudinal
Leite Faria et al. [70]	2009	Brazil	18830780	75	36.8 ± 10.7	80% women	43 ± 5.5	Food record (4 days)	None	RYGB	Assess postoperative eating patterns, relating them to weight loss.	Cross-sectional
Le Roux et al. [71]	2011	Sweden	21734019	16	N/D	69% women	N/D	Questionnaire	Yes	RYGB or VBG	Investigate how RYGB affects intake of and preference for high-fat food in an experimental (rat) study and within a trial setting (human).	Prospective
Ledoux et al. [72]	2017	France	27943093	78	43	81% women	44	Food record (4 days)	None	RYGB, SG or AGB	Explore whether self-reported preoperative changes in dietary habits and physical activity during a multidisciplinary preparation were predictive of postoperative weight loss.	Interventional
Magno et al. [73]	2014	Brazil	25409962	30	W: 48.4 ± 12.9; M: 49.8 ± 8.1	73% women	50.8 ± 14.5	24-h dietary recall	None	N/D	Evaluate the nutritional profile of the patients included into a multidisciplinary program for the treatment of severe obesity and bariatric presurgery.	Retrospective
Marin et al. [74]	2017	Brazil	28421792	45	20–45	100% women	Group 1: 47.8; Groupe 2: 41.5	Food record (3 days)	None	RYGB	Assess the effect of two micronutrient supplementation schemes on inflammation and iron metabolism in premenopausal women who had undergone RYGB surgery.	Prospective
Marques et Al. [75]	2020	Portugal	31435901	17	Symptomatic: 46.4 ± 1.7 Control: 42.1 ± 3.4	94% women	Symptomatic: 39.4 ± 1.8; Control 42.4 ± 1.2	Food and symptom diary (FSD)	None	RYGB	Evaluate the influence of meal nutritional composition on interstitial fluid glucose profiles and symptom profile after RYGB.	Cross-sectional
McLean et al. [76]	2018	United States	29100900	200	46.3 ± 8.5	100% women	48.9 ± 5.8	FFQ	None	RYGB, SG or LAGB	Identify usual dietary habits of black and white women seeking bariatric surgery and examine potential differences between these ethnic groups; to describe participants’plans to change dietary behaviors after surgery.	Cross-sectional
Melendez-Araùjo et al. [77]	2012	Brazil	23054569	32	39 ± 10.6	N/D	41.9 ± 5.2	24-h dietary recall	None	RYGB	Evaluate the impact of intensive and standard nutritional interventions on body weight, energy intake, and eating quality.	Retrospective
Melo et al. [78]	2017	Brazil	28724055	61	47.1 ± 9.9	84% women	31.5 ± 6.0	3 × 24-h dietary recall	None	RYGB or BPDS	Evaluate parameters of bone and mineral metabolism after bariatric surgery.	Sectional, retrospective
Mercachita et al. [79]	2014	Portugal	23955522	60	41.9 ± 12.2	65% women	42.3 ± 6.7	24-h dietary recall	None	RYGB	Quantify the intake of micronutrients in patients that were submitted to RYGB, determine the micronutrients deficiencies, and verify if the recommended vitamin and mineral supplementation intake would prevent theses deficiencies.	Retrospective longitudinal
Miller et al. [80]	2014	United States	24748474	17	47.3 ± 2.2	94% women	53.6 ± 1.7	Food record (4 days)	None	RYGB	Examine changes in macro- and micronutrients, food groups, and selected foods during 12-months of follow-up in post RYGB individuals.	Prospective
Mischler et al. [81]	2015	United states	26806728	36	45	97% women	32	Food record (3 days)	None	RYGB	Explore the impact of dietary and supplemental sources of iron and absorptive factors on iron status.	Cross-sectional
Moizé et al. [82]	2011	Spain	21298509	231	45.6 ± 9.9	72.3% women	48.2 ± 7.8	Food record (4 days) + 24-h dietary recall	None	N/D	Evaluate the dietetic intake and the prevalence of nutritional deficiencies in obese patients who are candidates for bariatric surgery.	Cross-sectional
Moizé et al. [83]	2013	Spain	23438491	355	SG = 46.4 ± 11.6; RYGB = 45.2 ± 10.6	75% women	SG = 51.6 ± 6.7; RYGB = 47.4 ± 6.0	Food record (3 days) + 24-h dietary recall	None	RYGB or SG	Prospectively compare dietary changes and nutritional deficiencies in grade 3 obese patients 5 years after SG and RYGB.	Longitudinal, prospective, observational
Molin Netto et al. [84]	2017	Brazil	27474230	41	39.4 ± 10.9	95% women	44.6 ± 6.3	FFQ	Yes	RYGB	Evaluate the early post-RYGB changes in the quality of eating patterns and their relationship to weight loss and metabolic parameters.	Longitudinal
Moore et al. [85]	2015	United-states	25270794	22	41± 12	100% women	46.7 ± 8	24-h dietary recall	None	RYGB (11) or SG (11)	Determine the response to 3 months of thiamin, B12, and folate supplementations.	Prospective observational
Nicoletti et al. [86]	2013	Brazil	21978750	80	45 ± 11	81% women	54 ± 8	24-h dietary recall	None	RYGB	Characterize the eating, anthropometric, and biochemical profile of obese candidates for bariatric surgery at a university hospital and assess their preoperative risk of nutritional deficiency.	Retrospective
Nicoletti et al. [87]	2015	Brazil	25851774	72	42 ± 9	86% women	53 ± 8	24-h dietary recall	None	RYGB	Evaluate the influence of red meat intolerance on the dietary pattern, biochemical indicators, and clinical symptoms after Roux-en-Y gastric bypass.	Retrospective
Nicoletti et al. [88]	2016	Brazil	27256164	150	47.2 ± 10.5	80% women	51.3 ± 7.3	24-h dietary recall	None	RYGB	Investigate the contribution of UCP2 gene variants on energy and macronutrients intake in a population after bariatric surgery.	Retrospective
Nicoletti et al. [89]	2020	Brazil	33231819	65	47.2 ± 11.4	86% women	35.5 ± 6.8	3 × 24-h dietary recall	None	RYGB or VBG	Investigate dietary habits and food intake during COVID-19 quarantine among patients who recently underwent bariatric surgery.	Cross-sectional
Nonino et al. [90]	2019	Brazil	31644673	441	44 ± 10	82.7% women	50.5 ± 8.0	24-h dietary recall	N/D	RYGB	Investigate nutritional status in 10 years follow-up.	Longitudinal retrospective
Nosso et al. [91]	2017	Italy	28969883	22	50 ± 9	54.5% women	31 ± 6	Food record (7 days)	None	RYGB (11) or SG (11)	Evaluate glycemic variability and oxidative stress in patients who achieved type 2 diabetes remission after bariatric surgery.	Cross-sectional
Novais et al. [92]	2012	Brazil	22652372	141	44 ± 9	100% women	45.9 ± 16.4	2 × 24-h dietary recall	Yes	RYGB	Assess the adequacy of food intake in women two or more years after bariatric surgery according to the excess weight lost.	Cross-sectional
Olbers et al. [93]	2006	Sweden	17060764	75	GB: 37.4 ± 0.4 VGB: 37.4 ± 0.5	50% women	GB: 42.3 ± 4.5; VBG: 42.6 ± 4.2	Questionnaire	Yes	RYGB(36) or VBG(39)	Evaluate the effect of dietary intake of on body composition and energy expenditure after sugery.	Prospective longitudinal
Ortega et al. [94]	2012	Spain	22722236	107	41.8 ± 9.8	79% women	50.7 ± 11.8	Food record (3 days)	None	RYGB	Analyze the likelihood of patients undergoing RYGB to recover a normal daily food intake, and the possible influence of dietary and exercise habits on long-term weight loss.	Cross-sectional
Papalazarou et al. [95]	2010	Greece	19834466	30	Usual care: 33.4 ± 2. Lifestyle intervention: 32.7 ± 1.6	100% women	Usual Care: 49.8 ± 1.6. Lifestyle intervention:48.5 ± 2.	24-h dietary recall	None	VBG	Evaluate the 3 year effects of a lifestyle intervention on weight loss and maintenance, dietary, and physical activity habits and eating behavior of patients following VBG.	Cross-sectional
Pinto et al. [96]	2019	Brazil	31376133	51	39.34 ± 9.38	68.7% women	43.0 ± 5.7	24-h dietary recall	None	RYGB	Evaluate changes in dietary intake and predictive factors of obesity remission in the first 12 months after RYGB.	Observational, prospective
Quesada et al. [97]	2014	Brazil	24724773	100	33.3 ± 6.08	100% women	45.75 ± 6.05	24-h dietary recall	Yes	Gastroplasty	Test 6 variations in the Goldberg equation to evaluate underreporting among obese women on a bariatric surgery waiting list.	Cross-sectional
Raatz [98]	2020	United States	32418771	72	44.1 ± 11.7	81% women	47.3 ± 6.9	2 × 24-h dietary recall	None	RYGB	Evaluate the reported macro- and micronutrient intake of adults who underwent RYGB over 7 years after surgery.	Longitudinal
Reid et al. [99]	2016	Canada	27744735	27	53.2 ± 8.3	89% women	33.8 ± 8.1	Food record (3 days)	Yes	RYGB	Compare the differences in dietary intake (caloric and macronutrient) between individuals who have maintained weight loss (maintainers) to those who have regained their lost weight (regainers) on average 12 years after RYGB and examine behaviours/habits between weight regainers and maintainers.	Retrospective
Ruiz-Lozano et al. [100]	2016	Spain	26948400	270	52 ± 11	82% women	46.5 ± 6	Food record (4 days)	None	RYGB (203) or SG (67)	Evaluate if food timing is associated with the weight loss effectiveness following bariatric surgery.	Observational
Ruiz-Tovar et al. [101]	2017	Spain	29250751	93	45.7 ± 10.8	78% women	46.4 ± 7.9	FFQ	None	SG	Evaluate the changes in the frequency intake of different foods in patients undergoing sleeve gastrectomy and following a strict dietary control.	Prospective, observational
Sanchez et al. [102]	2016	Chile	26108638	103	36 ± 9.6	100% women	43.1 ±5.3	FFQ	None	RYGB or SG	Evaluate dietary intake and nutritional status of various micronutrients in morbidly obese women prior to bariatric surgery.	Cross-sectional
Sarwer et al. [103]	2012	United-states	22551576	84	42 ± 9.9	63% women	51.6 ± 9.2	FFQ	Yes	RYGB (62) or AGB (16)	Evaluate the impact of dietary counselling on weight loss, dietary intake and eating behaviour after surgery.	Interventional
Sarwer et al. [104]	2008	United States	18586571	200	43.2 ± 9.8	82% women	52.1 ± 9.3	FFQ	Yes	RYGB	Investigate the relationship between preoperative eating behavior, postoperative dietary adherence and weight loss following gastric bypass surgery.	Prospective
Schoemacher et al. [105]	2019	the Netherlands	31313238	135	46.5 ± 9.5	83.7% women	44.6 ± 6.7	Food record (2 days)	None	RYGB or SG	Explore the relationship between total energy intake and % total body weight loss over a period of 4 years post-surgery.	Longitudinal, observational
Seki et al. [106]	2019	Japan	30711445	46	64.5 ± 8.1	47% women	31.7 ± 2.2	24-h dietary recall	None	DBP	Investigate the impact of metabolic surgery for diabetic patients with body mass index < 35 kg/m^2^ on health-related quality of life, food tolerance, and food satisfaction in a single institution.	Retrospective
Shah et al. [107]	2013	United States	24113734	23	49.3 ± 10.5	91% women	41.1 ± 6.2	Food record (3 days)	None	GB	Examine whether dietary counseling improves micronutrient and macronutrient intakes in GB surgery patients.	Prospective
Shai et al. [108]	2002	Israel	12568186	75	34.4 ± 9.4	81% women	41.4 ± 6.0	FFQ	None	VBG	Evaluate the long-term nutritional changes that occur in VBG patients compared with their nutrition before surgery.	Retrospective
Soares et al. [109]	2014	Brazil	24500225	172	42.4 ± 9.0	92.5% women	46.9 ± 6.0	FFQ	None	RYGB	Evaluate the life habits and diet quality of patients who have undergone bariatric surgery (who have been recovering for at least 6 months) based on the specific food pyramid.	Retrospective
Solga et al. [110]	2004	United-states	15573908	70	44 ± 9	89% women	55 (median)	24-h dietary recall	None	RYGB	Determine whether overall calorie intake and diet composition are associated with the severity of NAFLD histopathology.	Retrospective
Sovik et al. [111]	2013	Norway/Sweden	22951078	60	GB: 35.2 ± 7 DS: 36.1 ± 5.26	70% women	GB: 54.8 ± 3.24 DS: 55.2 ± 3.49	Food record (4 days)	None	BPD (29) or RYGB (31)	Evaluate the gastrointestinal side effects, caloric intake, and changes in obesity-specific quality of life 2 years after surgery.	Prospective longitudinal
Torres et al. [112]	2012	Brazil	22688468	44	45.4 ± 9.5	100% women	31.3 ± 4.8	Food record (4 days)	None	RYGB	Evaluate the nutrient intake of women who had undergone RYGB surgery.	Cross-sectional
Trostler et al. [113]	1995	Israel	10733792	55	RYGB: M: 41± 4/W: 32 ± 4 VBG: M: 32 ± 3/W: 37 ± 2	73% women	RYGB: 43 ± 4/W:43 ± 6 VGB: M:45± 7/W: 42± 8	FFQ + 24-h dietary recall	None	RYGB (19) or VBG (36)	Compare 2 surgeries with a low energy diet and dietary counseling. Compare the food intake pattern and nutritional composition of the food consumed over time.	Longitudinal
Ullrich et al. [114]	2013	Switzerland	22941334	44	N/D	N/D	47.3 ± 1.1	FFQ	None	RYGB	Investigate changes in the hedonic hunger and dietary habits after RYGB surgery	Longitudinal
Verger et al. [115]	2016	France	26205215	52	RYGB: 43.5; SG:41.0	67% women	RYGB: 45.5; SG:43.2	24-h dietary recall	Yes	RYGB (22) or SG (30)	Analyze food restriction effects on the nutritional adequacy of the diet, on macro- and micronutrient intake evolution, as well as their consequences in terms of bioclinical evolution and micronutrient serum level post surgery.	Retrospective
Vieira et al. [116]	2019	Brazil	30565102	40	stable weight 38± 7; weight regain 42 ± 11	100% women	SW 41.7 ± 6.5; WR 41.3 ± 3.5	24-h dietary recall + 2 food record (1 day)	None	RYGB	Investigate the perception of hunger and satiety and its association with nutrient intake in women who regain weight in the postoperative period after bariatric surgery.	Cross-sectional
Vieira et al. [117]	2020	Brazil	32022115	60	38.8 ± 9.6	78% women	47.3 ± 6.9	FFQ	None	N/D	Evaluate the association of food consumption with nutritional status, physical activity and sociodemographic factors in the bariatric surgery period preoperative	Cross-sectional
Vinolas et al. [118]	2019	France	31102207	57	RYGB: 42.9 ± 11 SG: 45.2 ± 9.2	N/D	RYGB: 46.8 ± 6.9 SG: 44.1 ± 9.4	Food record (7 days)	None	RYGB or SG	Evaluate nutritional status, micro- and macronutrient intake, and oral hydration in patients before and regularly during 1 year after RYGB and SG.	Retrospective
Wardé-Kamar et al. [119]	2004	United States	15479596	73	46 ± 11	93% women	54 ± 12	24-h dietary recall	None	RYGB	Investigate self-reported food intake, diet composition and meal patterns, in relation to long-term weight loss outcomes after RYGB.	Retrospective, longitudinal
Werling et al. [120]	2013	Sweden	23573244	14	GB: 59.7; VBG: 50.2	100% women	GB: 30.8; VBG: 35.0	Questionnaire	Yes	VGB or GB	Investigate alterations in postprandial EE after gastric bypass and VBG in humans.	Cross-sectional
Wolf et al. [121]	2015	Germany	25980331	43	44 ± 12	63% women	52.6 ± 10.5	Food record (3 days)	Yes	N/D	Assess the status of micronutrients in morbidly obese patients seeking bariatric surgery and to correlate extra-cellular nutrient levels with the corresponding nutrient intake.	Cross-sectional
Zaparolli et al. [122]	2018	Brazil	29972395	106	48 (20–64y)	90.5% women	39.6 (32.8–67.8)	24-h dietary recall	None	RYGB	Analyze food intake evolution during the first postoperative year of Roux-en-y gastric bypass in patients with type 2 diabetes or glycemic alteration.	Retrospective, longitudinal, observational
Ziadlou et al. [123]	2020	Iran	33046020	58	37 ± 8	71% women	44 ± 6	3 × 24-h dietary recall	None	RYGB or SG	Assess the adequacy of dietary nutrient intakes at 6th and 12th month after bariatric surgery.	Longitudinal

AA, African American; AGB, adjustable gastric banding; BI, bilio-intestinal bypass; BPD, Biliopancreatic diversion with duodenal switch; Ca, calcium; carb, carbohydrates; chol, cholesterol; Db, diabetic; DS, duodenal switch; eq, equivalent; FFQ, Food frequency questionnaire; GB, Gastric banding; GBP, Gastric bypass; MLVG, modified long vertical gastroplasty; NAFLD, non alcoholic fatty liver disease; nDb, non diabetic; N/D, not defined; RYGB, Roux-en-Y gastric bypass; Se, selenium; SG, Sleeve gastrectomy; TGVP, total gastric vertical plication; VBG, vertical banded gastroplasty.

**Table 3:** Validation of dietary assessment tools.

Author	Surgery Type	Reference Method	Validation			Conclusions About Validity
			Bariatric Population	Pre-and/or Post-Surgery	Directly in the Study	
**Food records (FR)**						
Bobbioni-Harsch et al. [27]	RYGB	Indirect calorimetry (resting energy expenditure; glucose, lipid and protein oxidation)	Yes	Pre-surgery	Yes	The degree of mis-report averages −17% of the evaluated energy requirements, in pre-surgery conditions; it represents a reasonable degree of inaccuracy [27].
Golzarand et al. [63]	RYGB or SG	Indirect calorimetry (resting metabolic rate, glucose, lipid and protein oxidation)	Yes	Pre- & post-surgery	Yes	In accordance with dietary intake reduction, protein and carbohydrate oxidation significantly decreased in both procedures post-surgery, while fat oxidation increased, but was not significant.
Reid et al. [99]	RYGB	9-days food record (energy, macro and micronutrients)	No		No	Relative validity of 3-days FR appears to be acceptable as dietary assessment tool [124].
Wolf et al. [121]	N/A	Correlation with vitamin A, D, E and C plasmatic values	Yes	Pre-surgery	Yes	No correlations were found between serum/plasma concentrations and nutritional intake nor associations between low concentrations and inadequate intakes.
**24-h dietary recall (24HR)**						
Aron-Wisnewsky et al. [23]	RYGB or AGB	24HR conducted by a dietitian (food consumption, energy and macro- and micronutrient intakes)	No		No	Agreement between the two methods was high, although it may have been overestimated because the two assessments were consecutives to one another. The tool may be highly advantageous for large population-based surveys [125].
Carvalho et al. [32]	RYGB or SG	Compared to Behavioral Risk Factor Surveillance System’s Fruit and Vegetable Consumption Module and the National Cancer Institute’s Percentage Energy from Fat Screener.	No		No	Validity of brief dietary intake measures may vary by demographic characteristics of the sample. Additional measurement work may be needed to accurately measure dietary intake in obese African-American women [126].
Kops et al. [67]	N/D	24-h urine sample (urinary urea to assess protein intake)	Yes	Pre-surgery	Yes	The 24HR was accepted as appropriate. Only 37.4% of patients gave an accurate record; another 37.4% underrreported, and 25.2% overreported.
Novais et al. [92]	RYGB	3-days FR (energy and nutrients)	Yes	Post-surgery	Yes	The agreement between the two methods (r = 0.91 to 0.98) evidenced low variability of the meals consumed by the group.
Quesada et al. [97]	GP	Indirect calorimetry (resting metabolic rate, energy requirement)	Yes	Pre-surgery	Yes	Comparing the results obtained for the modified Goldberg equations in this study, there was considerable variation in the proportion of underreporting (55% to 97%).
Verger et al. [115]	RYGB or SG	Indirect calorimetry (basal metabolic rate)	Yes	Pre- & post-surgery	Yes	Values revealed that patients from both groups underreported their caloric intake by 8% pre-surgery.
**Food frequency questionnaire (FFQ)**						
Amundsen et al. [18]	GB	Doubly labelled water (total energy expenditure)	No		No	The data showed that there was substantial variability in the accuracy of the FFQ at the individual level. Furthermore, the results showed that the questionnaire was more accurate for groups than individuals [127].
Benaiges et al. [26]	RYGB (43%)	3-day FR (dietary intakes)	No		No	A reasonable relative validity of the FFQ and 3-day FR for estimating nutrient intake was found [128].
Chou et al. [34]	SG	24HR (energy and macronutrients intakes)	Yes	Post-surgery	Yes	The energy intake according to the dietary questionnaire was 1230 kcal/day 5 years after LSG, and the 24HR method reported a daily energy intake of approximately 1083 kcal/day.
Farias et al. [50]	RYGB	3x 24HR (energy and macronutrients intakes)	No		No	Food consumption reports of overweight individuals tend to be underestimated. Despite its limitations, FFQ could be used in epidemiological studies to assess the regular food consumption of overweight individuals [129].
Johnson et al. [65]	RYGB	14-day FR (energy from fat and sugar) and correlation of fatty acids and Alpha-tocopherol in adipose tissue with serum	No		No	On average, 39% of the men were classified in the same quartile with the two methods, and 3% in the opposite quartile. Very-long chain n-3 fatty acids in adipose tissue and total serum lipids reflect the dietary intake of very-long-chain n-3 fatty acids to the same degree. No associations were observed between intake of alpha-tocopherol and concentration in adipose tissue and serum [130].
Molin Netto et al. [84]	RYGB	3 × 24HR (energy and macronutrients intakes)	No		No	Idem Farias et al. 2020 [129].
Sarwer et al. 2012 [103]	RYGB or AGB	4 and 7-day FR (energy and macronutrients intakes)	No		No	Correlations between questionnaire and FR for percent of energy from fat were 0.67 and 0.65 respectively in the two groups; most correlations were similar to those achievable by a single 4-day FR [131].
Sarwer et al. 2008 [104]	RYGB	4 and 7-day FR (energy and macronutrients intakes)	No		No	Idem Sarwer et al. 2012 [131].
**Questionnaires**						
Kanerva et al. [66]	LAGB or VBG or RYGB	4-day FR, 24-h energy expenditure and nitrogen excretion (nutrient intake, basal matabolic rate)	No		No	People with obesity reported energy and protein intakes 35% higher with the questionnaire compared with FR and nitrogen excretion [132].
Laurenius et al. [69]	RYGB	4-day FR, 24-h energy expenditure and nitrogen excretion (nutrient intake, basal matabolic rate)	No		No	Idem Kaverna et al. 2017 [132].
Le Roux et al. [71]	RYGB or VBG	4-day FR, 24-h energy expenditure and nitrogen excretion (nutrient intake, basal matabolic rate)	No		No	Idem Kaverna et al. 2017 [132].
Olbers et al. [93]	RYGB or VBG	4-day FR, 24-h energy expenditure and nitrogen excretion (nutrient intake, basal matabolic rate)	No		No	Idem Kaverna et al. 2017 [132].
Werling et al. [120]	GB or VBG	4-day FR, 24-h energy expenditure and nitrogen excretion (nutrient intake, basal matabolic rate)	No		No	Idem Kaverna et al. 2017 [132].
**Other dietary assessment methods**						
Al-Ozairi et al. [17]	SG	24HR by a dietitian (energy, macronutrients, fiber, total fat, saturated fat, mono- polyinsaturated fat, cholesterol and sodium)	Yes	Post-surgery	Yes	After SG, patients reported higher total energy intake and energy intake from carbohydrates compared to estimations using photographs. Digital photography appears reliable and accurate in adults in measuring energy intake in a cafeteria setting.
**Mixed methods**						
Brolin et al. [28]	VBG or RYGB	1 week FFQ (energy, protein, carbohydrate and fat intake)	Yes	Pre- & post-surgery	Yes	Multiple tools were used to obtain a mean of energy intake and macronutrients.
Casagrande et al. [33]	RYGB	FFQ + 24HR (total energy, macro and micronutrients)	Yes	Pre-surgery	Yes	The FFQ underestimated total energy value intake as compared with the 24HR. Protein and lipid intakes were lower if evaluated by the FFQ as compared to the 24HR. Calcium intake was higher when evaluated by the FFQ as compared with the 24HR.
El Labban [47]	RYGB or SG	N/D	No		No	N/D

AGB, adjustable gastric banding; BDP, biliopancreatic diversion with duodenal switch; FFQ, food frequency questionnaire; GB, Gastric banding; GP; gastroplasty; kcal, Kilocalories; LAGB, Laparoscopic Adjustable Gastric Banding; N/D: Not defined; RYGB, Roux-en-Y gastric bypass; SG, Sleeve gastrectomy; VBG, vertical banded gastroplasty; 24HR, 24-h recall.
